# Automatic capture of attention by flicker

**DOI:** 10.3758/s13414-020-02237-2

**Published:** 2021-02-19

**Authors:** Moritz Stolte, Ulrich Ansorge

**Affiliations:** 1 Faculty of Psychology, University of Vienna, Liebiggasse 5, A-1010 Vienna, Austria; 2 Vienna Cognitive Science Hub, University of Vienna, Vienna, Austria; 3 Research Platform Mediatised Lifeworlds, University of Vienna, Vienna, Austria

**Keywords:** attentional capture, automaticity, Motion

## Abstract

Visual motion captures attention, but little is known about the automaticity of these effects. Here, we tested if deviant flicker frequencies, as one form of motion, automatically capture attention. Observers searched for a vertical target among tilted distractors. Prior to the target display, a cue array of sinusoidally modulating (flickering) annuli, each surrounding one location of the subsequent target(-plus-distractors) display was presented for variable durations. Annuli either flickered all at 1 Hz (neutral condition, no-singleton cue), or a single annulus flickered at a unique frequency of 5 Hz, 10 Hz, or 15 Hz. The location of this singleton-frequency cue was uncorrelated with target location. Thus, we could measure benefits (target at cued location) and costs (target ≠ cued location) for cues of different frequencies and durations. The results showed that deviant flicker frequencies capture attention, as we observed benefits and costs, falsifying that nonspatial filtering accounted for the cueing effect. In line with automatic capture, cueing was effective in singleton (Experiment 1) and nonsingleton search tasks (Experiment 2), and is thus not dependent on (“top-down”) singleton detection mode. Moreover, analysis of results ruled out trial-by-trial “swapping” of flicker frequencies from preceding target to subsequent distractor locations. Results also revealed increasing cueing effects with higher cue flicker frequency and longer duration. This indicates a significantly longer period of automatic capture by sinusoidal flicker than the typical inhibition of return observed around 250 ms after the onset of uninformative static or single-transient cues.

**Supplementary Information:**

The online version contains supplementary material available at 10.3758/s13414-020-02237-2.

## Introduction

At each moment in time, humans select only a fraction of the available information from their surrounding visual environment (Driver, 2001). This is called visual attention. One important driver of visual attention is top-down goals (Folk, Remington, & Johnston, 1992). For example, humans can actively search for relevant visual targets by some of their features and successfully ignore irrelevant distractors, especially if there are large feature differences between target and distractors (Duncan & Humphreys, 1989; Wolfe, 2018). However, if visually salient, distractors can capture attention in a bottom-up manner (Itti, Koch, & Niebur, 1998). For example, distractor singletons, standing out by a unique color among nonsingletons of a different color, reliably attract attention during visual search for other feature targets and, thereby, delay search (Theeuwes, 1992).

A particularly interesting case of attentional capture, due to its often highly salient features and abundance in everyday life, is visual dynamics. Intuitively, local changes—that is, visual motion, in the visual environment seem to attract attention in a bottom-up manner. In line with this, early research showed that humans are unable to ignore abrupt onsets—the sudden appearance of a stimulus in the field of view (Yantis & Jonides, 1984). Over time, this view gave way to an alternative perspective: contingent-capture theory (Folk et al., 1992). According to contingent-capture theory, visual dynamics capture attention only if participants actively search for dynamics. For example, an abrupt-onset singleton distractor captures attention when humans search for abrupt-onset targets, but not when they search for static features, such as color-defined targets (Folk et al., 1992). However, more recent evidence is increasingly supporting the claim that salient visual dynamics can summon attention in a bottom-up fashion, even during search for static features (Gaspelin, Ruthruff, & Lien, 2016; Schoeberl, Fuchs, Theeuwes, & Ansorge, 2014).

One very interesting visual dynamic is flicker (Cass, Van der Burg, & Alais, 2011; Spalek, Kawahara, & Lollo, 2009). Visual flicker is used, for example, in police or ambulance flashing lights, and it can resemble abrupt onsets, in that flicker consists of several abrupt onsets following one another at the same location. What distinguishes processing of flicker from onsets, though, is the duration necessary to discriminate between flicker and onsets or other motion stimuli (e.g., different flicker rates). For example, to discriminate a singleton flicker frequency from surrounding nonsingleton frequencies, humans would have to integrate visual information across time. In contrast to abrupt onsets, salient flicker could therefore theoretically capture or engage visual attention for extended durations, whereas the brief duration of abrupt onsets implies that their effects are short lived and quickly give way to passive decay (cf. Donk & van Zoest, 2008) or active suppression (cf. inhibition of return or IOR; Mulckhuyse, Talsma, & Theeuwes, 2007; Posner & Cohen, 1984).

Importantly, whether flicker attracts attention automatically or whether attention capture depends on top-down factors has not been clearly established. For other distractors such as onsets or static cues, different protocols have yielded contradicting results. Whereas the additional singleton protocol (where a singleton distractor is presented concomitantly with nonsingleton and target stimuli; e.g., Theeuwes, 1992) has consistently shown automatic capture of attention by task-irrelevant features, the contingent-capture protocol (where cues appear before the target-search displays; e.g., Folk et al., 1992) in contrast, has consistently produced the opposite result—no capture of attention by cues that do not match the top-down control settings to search for the targets. Previous research on attentional capture by flicker used a variant of the additional singleton paradigm protocol (Cass et al., 2011).

Here, we used a variant of the contingent contingent-capture protocol to test whether a deviant flicker cue can break through the top-down set and inevitably capture attention automatically during search for static targets. We based our protocol on that of Experiment 2 in Cass et al. (2011), the results of which suggested automatic capture of attention by a deviant singleton-flicker cue presented during search for static targets. However, we made two decisive changes to the protocol. Firstly, instead of presenting the cue as an additional singleton, we presented the cue within a cueing display preceding the target search display by up to 380 ms. Secondly, we controlled for alternative accounts, such as trial-by-trial priming and feature swapping: Across trials, Cass et al. (2011) randomly selected flicker frequencies at target and distractor locations to the effect that their frequencies were sometimes swapped, meaning the flicker frequency at target position in trial *n* was used as the frequency at a distractor location in an immediately following trial *n* + 1. To test whether flicker singletons capture attention in a bottom-up way, these conditions are not ideal, as priming of capture could explain the results equally well. The fact that participants attended to the target implies that features present at target location might have primed capture by similar features in an immediately following trial (and maybe even beyond; cf. Maljkovic & Nakayama, 1994). Here, to prevent priming, we used singleton-frequency distractors as cues that were not predictive of target positions across trials in that they were presented at target location (as valid cues) or away from the target (as invalid cues), but made sure that distractor benefits (faster RTs on valid compared with neutral trials) and costs (slower RTs on invalid compared with neutral trials) in trial *n*, were reliably observed even when an invalid cue was used in trial *n −* 1. Note that on successful invalid trials, participants had attended to the distractor frequency before attention was shifted to the target. Thus, by demonstrating reliable cueing benefits and costs for trials following invalid trials, feature swapping and priming of a current trial’s cue features relative to preceding trial’s target features were ruled out.

If salient singleton frequencies capture attention in an automatic, bottom-up way, a singleton-frequency distractor presented away from target position should interfere with target search. This should show up as a cost—slower search times in trials with an invalid singleton-frequency distractor compared with neutral trials (without a singleton distractor). Likewise, a valid singleton-frequency distractor presented at target position should lead to a benefit—facilitating search relative to neutral trials. If deviant flicker frequencies do not automatically capture attention (in line with contingent-capture theory), no differences in search times between valid or invalid trials compared with neutral trials should be observed.

## Method

### Participants

Twenty-four observers (nine males, 15 females) between 19 and 35 years of age (*Mdn* = 23 years, *SE* = 1.12), participated in Experiment 1, and 24 new observers (eight males, 16 females) between 21 and 36 years of age (*Mdn* = 26.5 years, *SE* = 0.88) participated in Experiment 2. All participants were recruited from the University of Vienna’s subject pool, reported normal or corrected-to-normal vision, signed informed consent before participating, and received course credits for completing the experiment.

### Stimuli and apparatus

**Fig. 1:**
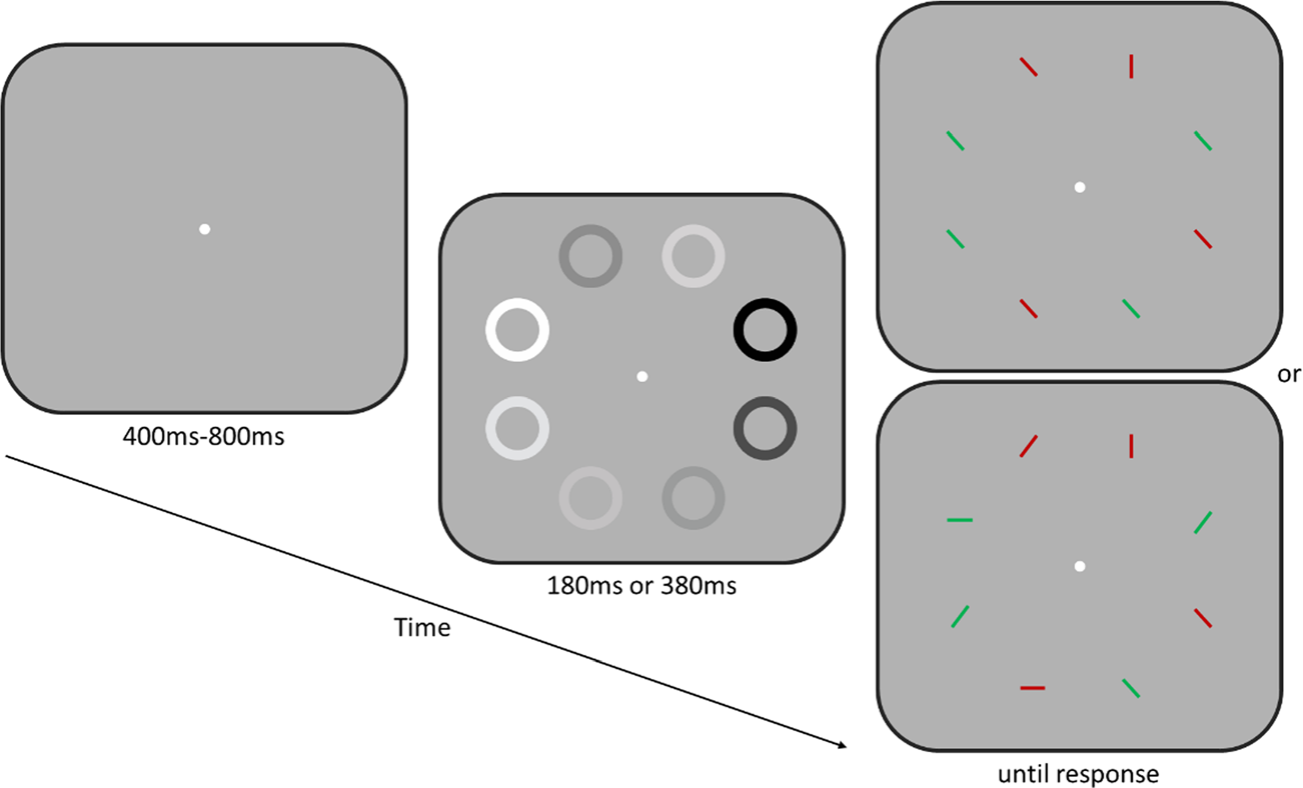
Illustration of the sequence of events for trials in Experiment 1 (top right display) and Experiment 2 (bottom right display). After a variable fixation duration, annuli were presented at eight locations and flickered sinusoidally around mean luminance. On half of the trials, the speed of flicker of a frequency-singleton cue (5 Hz, 10 Hz, or 15 Hz) differed from the nonsingleton annuli (1 Hz), while all annuli flickered at the same speed (1 Hz) in the remaining (i.e., neutral) trials. The cue display containing the flickering annuli was presented for either 180 ms or 380 ms. The subsequent target-search display remained on screen until a response was made. Participants indicated the color (green or red) of a single vertical line among homogeneous (Experiment 1) or heterogeneous (Experiment 2) distractor lines

Experiments were run in a dimly lit room. Participants sat ~50 cm from an LCD monitor with a 100 Hz refresh rate (luminance linearized by gamma correction). To maintain viewing distance, participants’ heads were fixated with a chin and forehead rest. All stimuli were presented on a medium gray background. A cue display preceding the target display consisted of eight annuli (1.1° radius, 0.4° width), one surrounding each stimulus location (see Fig. 1). The luminance of each annulus varied sinusoidally over time around mean luminance (gray). Moreover, in order to minimize luminance cues that can occur as a function of stimulus frequency (Bex & Langley, 2007; de Lange, 1958) and may affect visual search, the modulation depth of each individual annulus (Lmax − Lmin/Lmax + Lmin) was randomly selected from a range of ±20% around an average modulation depth of 77%. The starting phase of each temporal modulation was also randomized to exclude predictable phase changes and resulting grouping effects (Spalek et al., 2009).

Half of the trials (768/1,536) involved cue annuli all flickering at the same frequency (1 Hz). These trials we refer to as the neutral condition (neutral cueing, 50% of all trials). Other trials consisted of a singleton annulus flickering at a unique frequency (5 Hz, 10 Hz, or 15 Hz), while the nonsingletons always flickered at 1 Hz. Most frequently (probability of 7/8, total 672 trials), this singleton frequency was equally likely at each of the distractor locations (invalid cueing, 43.75% of all trials). In the remaining 1/8 of all trials, the singleton annulus surrounded the subsequent target location (valid cueing, 6.25% of all trials). Participants took short breaks (<1 min) and received feedback about mean accuracy and mean reaction time (RT) following each block (24 in total). Target displays following the cues consisted of four red (CIE L*a*b* coordinates, 38.7/63.3/49.6) and four green (52.9/−51.4/32.9) line segments (length 0.7° visual angle) presented on the same gray mean-luminance background (56.1/−0.7/−1.5) as the cues. Lines were equally spaced on an imaginary circle (4.9° radius) centered on a white fixation dot. Line orientations were vertical, horizontal, or diagonal (45° to the left or right from vertical).

### Design and procedure

The procedure was identical in both experiments, except for the distractor items in the stimulus displays. The target was always a single vertical line. In Experiment 1, all distractors were tilted 45° to the left from vertical. In Experiment 2, a random half of the trials had stimulus displays identical to those in Experiment 1 while in the rest of the trials stimulus displays contained heterogeneous distractors: 2–3 horizontal, right-tilted or left-tilted lines each. Thus, singleton detection mode (i.e., “top-down” search for singletons that differ from their background) was possible in Experiment 1 but not in Experiment 2 (cf. Bacon & Egeth, 1994).

The participants’ task was to search for and indicate via keypress as quickly and accurately as possible the color of the vertical target line (red or green). Correct RTs and target accuracy rates were measured. Each trial began with a blank screen (250 ms) followed by a central fixation dot presented for a variable duration (randomly chosen between 400 ms and 800 ms). Then the cue display was presented for either 180 ms or 380 ms, immediately followed by the target-search display, which remained on screen until a response was made (see Fig. 1). In both experiments, the cues disappeared with target-search display onset. Participants were instructed that cue locations (i.e., the location of the singleton-frequency annulus) were random and had no relationship to target locations. They were also asked to maintain fixation on the central fixation point throughout the experiment.

## Results

### Reaction time

Mean RTs from correct trials of both experiments were entered into a single repeated-measures analysis of variance (ANOVA), with within-participant independent variables cue type (valid, invalid), cue duration (180 ms, 380 ms), and cue frequency (5 Hz, 10 Hz, 15 Hz) and between-participants variable experiment. Trials with RTs shorter than 200 ms or exceeding 2 s were excluded from analysis (<0.8% of all trials).

The ANOVA revealed a significant three-way interaction between cue duration, cue type, and cue frequency, *F*(2, 92) = 3.90, *p* < .05, η_p_^2^ = .078. However, the between-participants variable experiment did not interact with any of the other factors (all *p*s > .11), indicating a similar pattern of results across the two experiments. Overall performance was slightly better in Experiment 1 (*M* = 636 ms, *SE* = 22.12) than Experiment 2 (*M* = 705 ms, *SE* = 22.12), *F*(1, 46) = 4.89, *p* < .05, η_p_^2^ = .096.

Please note, additional information is presented in the Supplementary Material: mean RTs and accuracies are shown in Tables S1–S2 and means for Experiments 1 and 2 are reported separately in Tables S4–S5 as well as in Figs. S1 and S2. In addition to the ANOVAs presented below, exploratory *t* tests against zero were conducted for each cue frequency and duration in order to see if these more fine-grained tests supported the conclusions drawn from the ANOVAs. Individual comparisons were always corrected using Bonferroni, except for the *t* tests of cueing effects, cueing costs, and cueing benefits against zero, where a less conservative correction was applied (Benjamini & Hochberg, 1995).

**Fig. 2:**
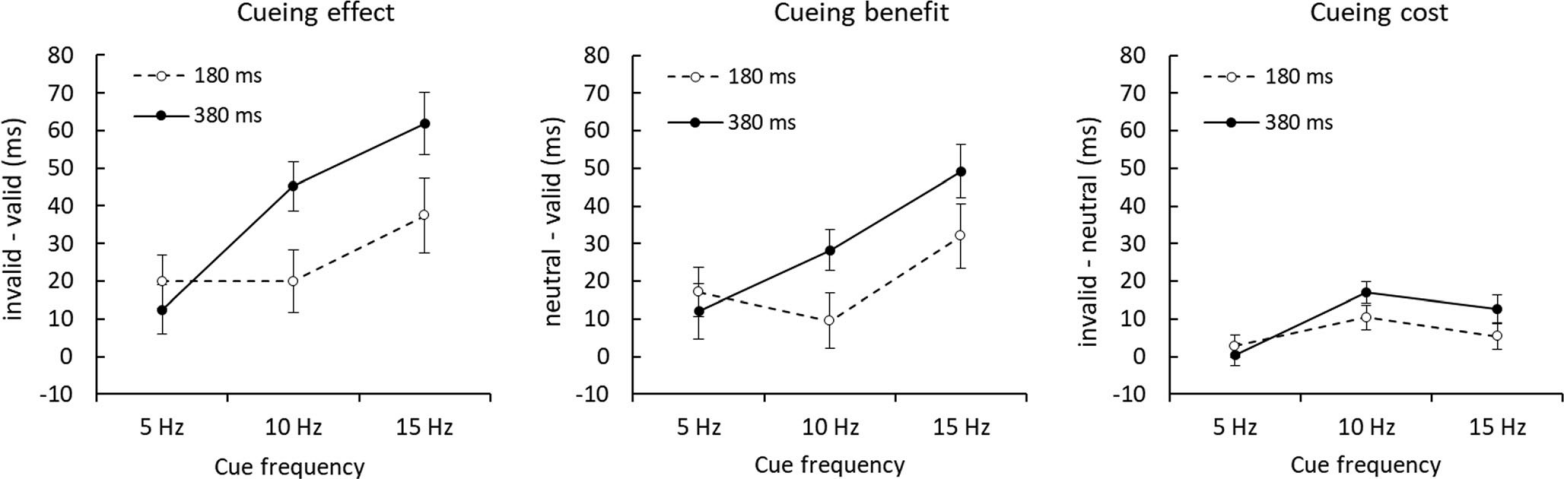
RT cueing effects (invalid minus valid, left panel), cueing benefits (neutral minus valid, central panel), and cueing costs (invalid minus neutral, right panel) as a function of the cue frequency (5 Hz, on the left; 10 Hz, in the center; 15 Hz, on the right of each respective panel), and duration (180 ms, broken lines; 380 ms; straight lines), with data averaged across Experiments 1 and 2. We observed both, significant benefits and costs at 10 Hz and 15 Hz cue frequencies. Error bars represent ±1 *SE*

Separate repeated-measures ANOVAs were conducted for cueing effects (invalid – valid RTs), benefits (neutral – valid RTs), and costs (invalid – neutral RTs). The ANOVA on cueing effects, with independent variables cue duration (180 ms, 380 ms) and cue frequency (5 Hz, 10 Hz, 15 Hz), revealed a significant interaction between duration and frequency, *F*(2, 94) = 3.85, *p* < .05, η_p_^2^ = .076, indicating a linear increase in cueing from 5 Hz (*M* = 12.49 ms) to 10 Hz (*M* = 45.28 ms) and 15 Hz (*M* = 61.88 ms), in the long cue duration (380 ms) condition (all *p*s < .05, for comparisons between all three frequencies; Bonferroni corrected for multiple comparisons), while in the short cue duration (180 ms) condition, there were no significant differences for the magnitude of the cueing effect between the three cue frequencies. Despite employing completely uninformative cues, cueing was effective in reducing RTs (compared with invalid cues) in all conditions, except for 5 Hz cues in the long duration condition (see Table 1 for *t* tests against zero; Fig. 2, left). The analysis for benefits revealed a main effect of frequency, *F*(2, 94) = 7.91, *p* < .01, η_p_^2^ = .14, indicating stronger cueing benefits at 15 Hz than 5 Hz and 10 Hz (*p*s < .01). Cueing benefits were not significant at 10 Hz for the short cue duration (180 ms) and at 5 Hz for the long cue duration (380 ms). Additionally, a repeated-measures ANOVA on costs revealed a significant effect of frequency, *F*(2, 94) = 7.36, *p* < .01, η_p_^2^ = .16, with higher costs for 10 Hz (*M* = 13.71 ms) than 5 Hz (*M* = 1.63 ms) cues (*p* < .01), but no effect of duration and no interaction (*p*s > .08). Moreover, significant costs were observed for 10 Hz cues (for both cue durations, *p*s < .05) and 15 Hz cues (in the long cue duration condition only, *p* < .05; see Table 1).

**Table 1:** Cue validity effects (invalid − valid), cueing benefits (neutral − valid) and cueing costs (invalid − neutral) on reaction times as function of cue duration (180 ms, 380 ms) and frequency (5 hz, 10 hz, 15 hz) averaged across Experiments 1 and 2 (*df* = 47)

	Duration (ms)	Frequency (Hz)	RT Δ (ms)	*SE*	*t* (vs. 0)	Cohen’s *d*
**Cueing effect** (invalid − valid)	180	5	19.89	6.99	2.85*	0.41
		10	19.93	8.30	2.40*	0.35
		15	37.45	9.85	3.80**	0.55
	380	5	12.49	6.58	1.90	0.27
		10	45.28	6.54	6.92***	0.99
		15	61.88	8.20	7.55***	1.09
**Cueing benefit** (neutral − valid)	180	5	17.09	6.56	2.59*	0.38
		10	9.51	7.39	1.29	0.19
		15	32.04	8.49	3.77***	0.54
	380	5	12.02	7.25	1.66	0.24
		10	28.28	5.42	5.22***	0.75
		15	49.23	7.07	6.97***	1.01
**Cueing cost** (invalid − neutral)	180	5	2.79	3.01	0.93	0.13
		10	10.43	3.28	3.18*	0.46
		15	5.41	3.49	1.55	0.50
	380	5	0.47	2.95	0.16	0.02
		10	17.00	2.93	5.80***	0.84
		15	12.66	3.81	3.32**	0.48

*Note. p* < .05; **p < .01; ***p < .001. Corrected for multiple comparisons (Benjamini & Hochberg, 1995).

### Accuracy

Accuracy rates were largely at ceiling in both experiments. On average, participants responded correctly on 97% (*SE* = .86) of valid trials and 96.1% (*SE* = .65) of invalid trials in Experiment 1, and 96% (*SE* = 1.10) of valid and 95.7% (*SE* = .74) of invalid trials in Experiment 2. A repeated-measures ANOVA, with within-participant independent variables cue type, cue duration, and cue frequency and between-participants variable experiment on accuracy rates, showed no significant effects or interactions (all *p*s > .06). Separate ANOVAS for cueing effect (valid – invalid) and cueing benefit (valid – neutral) also showed no significant interactions. For cueing costs (neutral – invalid), however, the ANOVA on accuracy scores revealed a marginally significant interaction between cue duration and frequency, *F*(2, 94) = 3.01, *p* = .054, η_p_^2^ = .06, but no significant main effects. The interaction indicated increasing cueing costs from 5 Hz (*M* = −0.05%, *SE* = 0.25) to 10 Hz (*M* = 0.64%, *SE* = 0.39) and 15 Hz (*M* = 1.16%, *SE* = 0.43) cues for the long cue duration (380 ms), while for the short cue duration (180 ms) cueing costs remained constant for the different cue frequencies.

#### Additional analysis excluding trials where the singleton frequency cue changed from the target position on trial n − 1 to distractor position on trial n

Here, we separately analyzed cueing costs and benefits for trials immediately following invalid trials. Finding significant costs in these trials alone rules out feature swapping and intertrial priming accounts for the cueing effects and instead suggests involuntary shifts of spatial attention to the cued location as the most likely mechanism. Cueing costs alone (in comparison to neutral cues), however, could also be explained in terms of general filtering of any irrelevant objects that simply slows the deployment of spatial attention to the target location, but does not attract spatial attention per se. If such a filtering strategy was employed in the current study, we would expect no cueing benefits (compared with neutral trials) as slowing would occur on every trial, independent of the cue location. The results, however, show larger cueing benefits compared with costs and thus further support the role of spatial shifts of attention. Also note how the following analysis equates the influences of suppressed nonsingleton features (cf. Kristjánsson & Driver, 2008): In all trials for the analyses of both, cueing costs and benefits, the frequency at the target location on trial *n* − 1 was never different from the frequency at the nonsingleton distractor locations on trial *n*. Thus, trial-by-trial buildup or carryover of suppression of irrelevant nonsingleton features could not have contributed to either costs or benefits.

**Fig. 3:**
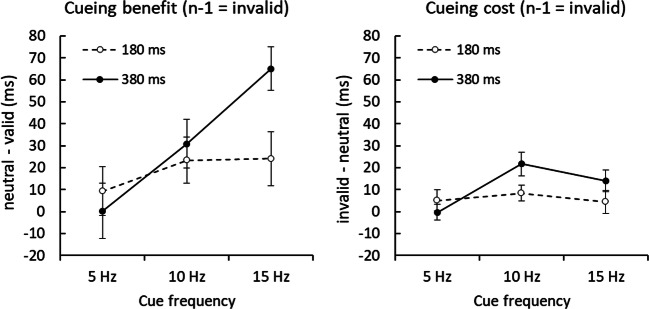
RT cueing benefits (neutral minus valid, left panel) and cueing costs (invalid minus neutral, right panel) as a function of the cue frequency (5 Hz, on the left; 10 Hz, in the center; 15 Hz, on the right of each respective panel), and duration (180 ms, broken lines; 380 ms; straight lines), with data averaged across Experiments 1 and 2, for trials following an *n* − 1 invalid trial only. Cueing benefits (neutral − valid) were larger than cueing costs (invalid − neutral) in these trials. Error bars represent ±1 *SE*

We separately analyzed cueing costs (invalid – neutral RTs) in all trials that were preceded by invalid trials only (thus, excluding all trials where target features were “swapped” to distractor locations; see Fig. 3; Table 2). A repeated-measures ANOVA as above, but now on trials following an invalid trial (*n −* 1 = invalid) showed a significant main effect of frequency, *F*(2, 92) = 3.57, *p* < .05, η_p_^2^ = .07, and a significant interaction between cue duration and frequency, *F*(2, 92) = 1.58, *p* < .05, η_p_^2^ = .06. RT cueing costs in these trials were significant at 10 Hz cue frequency for the short cue duration (*p* < .05) and at 10 Hz and 15 Hz cue frequencies for the long cue duration (*p* < .001 and *p* < .01, respectively). The same ANOVA was performed on accuracy scores, which showed no significant interactions. However, accuracy costs were significant at 15 Hz cue frequency for the long cue duration only, *t*(47) = 2.78, *p* < .01, *d* = .40. Thus, significant costs were still observed when all trials where swapping of target and distractor features could have occurred were excluded.

**Table 2:** Cueing benefits (neutral − valid) and cueing costs (invalid − neutral) in reaction times as function of cue duration (180 ms, 380 ms) and frequency (5 hz, 10 hz, 15 hz) for all trials preceded by invalid trials (*n* − 1 = invalid) with data averaged across Experiments 1 and 2 (*df* = 47)

*n* − 1 invalid	Duration (ms)	Frequency (Hz)	RT Δ (ms)	*SE*	*t* (vs. 0)
**Cueing benefit** (neutral − valid)	180	5	9.31	11.18	.83
		10	23.43	10.58	2.21*
		15	24.16	12.26	1.97
	380	5	0.33	12.65	.03
		10	30.91	11.18	2.77*
		15	65.11	9.93	6.55***
**Cueing cost** (invalid − neutral)	180	5	5.07	4.86	1.04
		10	8.44	3.72	2.27*
		15	4.41	5.28	.84
	380	5	−0.25	3.73	−0.07
		10	21.67	5.49	3.95***
		15	13.90	4.99	2.79*

*Note.* *p < .05; **p < .01; ***p < .001. Corrected for multiple comparisons (Benjamini & Hochberg, 1995)

The same analysis was performed for cueing benefits (neutral – valid RTs) for those trials preceded by invalid trials, showing a significant main effect of frequency, *F*(2, 92) = 6.27, *p* < .01, η_p_^2^ = .12. RT benefits in these trials were significant at 10 Hz in both cue duration conditions, *t*(47) = 2.21, *p* < .05, *d* = .32; and *t*(47) = 2.77, *p* < .05, *d* = .40, short and long, respectively; as well as, at 15 Hz in the long cue condition, *t*(47) = 6.55, *p* < .001, *d* = .95. Analysis of the accuracy scores for the same trials showed no significant interactions. However, cueing benefits in accuracy were significant at 15 Hz for the short cue duration, *t*(47) = 1.82, *p* < .05, *d* = .26.

Finally, in trials preceded by invalid trials, overall RT cueing benefits (*M* = 25.5 ms, *SE* = 11.3 ms) were larger than cueing costs (*M* = 8.9 ms, *SE* = 4.7 ms; see Table 2; Fig. 3). An ANOVA, with variables cueing effect type (benefits vs. costs), cue duration, cue frequency and experiment, confirmed significantly higher benefits compared with costs, *F*(1, 46) = 10.16, *p* < .01, η_p_^2^ = .18. In addition, there was a significant interaction between cueing effect type (benefits vs. costs) and cue frequency, *F*(2, 92) = 3.37, *p* < .05, η_p_^2^ = .07, indicating increased cueing benefits with increasing cue frequencies while cueing costs peaked at 10 Hz and decreased again at 15 Hz.

These results demonstrate that (1) significant search costs (invalid – neutral RTs) are still observed in trials where no feature swapping occurred, and (2) cueing benefits (neutral – valid RTs) in trials following invalid trials are significantly larger than cueing costs (invalid – neutral RTs). The significant costs in trials immediately following invalid trials rule out feature swapping and inter-trial priming accounts for the cueing effects and instead suggest involuntary shifts of spatial attention to the cued location as the most likely mechanism.

## Discussion

The current study demonstrates that flicker singletons capture attention in automatic, bottom-up fashion. The singleton-frequency cue was entirely uninformative of target locations or features. It did not match the search templates for a static orientation target, and the target was also not a singleton in Experiment 2. Thus, contingent capture based on singleton detection mode was ruled out. Finally, the capture effect was found even following *n* − 1 invalid trials. Therefore, the cuing effect was not restricted to trials in which a frequency at target position was used for a singleton distractor in the immediately following trial, thereby, ruling out an inter-trial priming account for the effect.

The results suggest, in contrast to contingent-capture theory, that deviant singleton-flicker frequencies can break through the top-down search set and capture attention. Folk and Remington (2015) have demonstrated similar results for rare onset cues, indicating that at least rare onsets can outweigh the top-down task-set. However, it was later shown that even the automatic capture effects of rare onsets can be attenuated when the task set is optimized to include precise suppression of nontargets. If a nontarget (presented together with the target in the search display) contains the cue properties (e.g., the color of the onset cue), the cue can be sufficiently suppressed and does not capture attention (Schoenhammer & Kerzel, 2018). Further research is needed to test if a precise top-down set that includes suppression of nontarget features can also prevent stimulus-driven capture by flicker-frequency singletons.

While the results seem to be at odds with contingent-capture theory (Folk et al., 1992), they also do not fit well to models assuming that visual search can be supported by the successful suppression of irrelevant signals (e.g., Kerzel & Barras, 2016; Sawaki & Luck, 2010; but see Gaspelin & Luck, 2018, for a review of the theory that argues that signal suppression may not apply to dynamic stimuli) or to models implying that search is directed to target–distractor differences (Becker, 2010; Navalpakkam & Itti, 2007). We think that the human visual system may be relatively sensitive to flicker as one form of visual change or motion signal associated with potentially novel input (cf. von Mühlenen, Rempel, & Enns, 2005; Yantis & Hillstrom, 1994) and that this signal is comparatively stronger than a single onset or translational motion signal, as translational motion only seems to capture attention in an automatic manner under particular, more restricted conditions (cf. Franconeri & Simons, 2003).

From the point of view of guided search theory (Wolfe, 2018) or from a priority map perspective (Fecteau & Munoz, 2006), an overall map of candidate regions for an attention shift may receive input from several feature maps that work relatively independent from one another, including static feature maps and motion-sensitive, dynamic feature maps. While, in the current study, it would be relatively easy to distinctively weight static target features versus distractor features (here, line orientations) in a top-down way for guided search, this would be relatively difficult for the dynamic feature of flicker which may only be differentiated by its frequency from all other visual dynamics (including the onsets of the targets). This idea is reminiscent of the display-wide contingent-capture theory according to which any target-associated feature could receive some top-down weight, even if it comes at the expense of allowing a degree of attention capture by irrelevant distractors presented away from the target (Burnham, 2007; Gibson & Kelsey, 1998). In this sense, a flicker stimulus could be a “super-stimulus” variant of the target-associated abrupt onset—that is, the flicker could be a particularly strong version of an abrupt onset which would usually, however, be more actively controlled by timed counteracting suppression in order to keep its influence on performance in check during search for targets.

The current results, with numerically relatively similar stimulus-driven cueing effects in less difficult search conditions (Experiment 1) and in more difficult search conditions (Experiment 2) appear at odds with previous findings (Gaspelin et al., 2016). These authors observed evidence for stimulus-driven capture by an onset cue only during search for color-defined targets, where target color and colors of distractors in the target display were relatively similar and, thus, search was difficult. In contrast, they observed no stimulus-driven capture by onset cues in a condition where target color and distractor color were more dissimilar, and search was easier. The authors argued that attention only dwelled long enough for a cueing effect at the cued location in difficult search conditions. A number of possible differences come to mind when comparing the present study with that of Gaspelin et al. (2016), such as search for orientations in the present study versus search for color in theirs, or a manipulation of search difficulty via target-distractor similarity in their study versus via distractor-homogeneity/heterogeneity during target search in the present study. Most critically, however, the present study’s flickering cues might have simply provided a stronger signal than a single onset. In fact, flicker can be conceived of as a repetition of onsets, leading to more frequent stimulation of the corresponding sensory detectors compared with a single onset. Maybe this stronger signal of the cue created the overall stronger capture effect which also led to a significant cueing effect in the easier search condition. A tendency for the cueing effects to increase with flicker frequency in the current study is certainly in line with this possibility. However, future research is needed to establish if higher flicker frequencies or larger singleton-flicker-to-nonsingleton-flicker differences account for the size of the cueing effects.

Attentional capture by flicker-singletons was spatial in nature, as was evident by significant costs in invalid trials and significant benefits in valid trials (both relative to neutral trials). Although invalid cue trials were frequent (43.75%), while valid cue trials were rare (6.25%), the presence of the deviant cue frequency in invalid trials (compared with neutral cue trials containing no cue; 50%) resulted in significant costs.^1^ Therefore, the cueing effect likely reflected a spatial effect such as shifting of attention (Folk et al., 1992) or postperceptual biases to report from the cued location (Shiu & Pashler, 1994) rather than nonspatial filtering costs (cf. Folk & Remington, 1998).

Furthermore, attentional capture effects were stronger with larger singleton-nonsingleton frequency differences and longer cue durations. The cueing effect was significantly stronger for 15 Hz and 10 Hz singleton frequencies than for 5 Hz singleton frequency. Although this could mean that the cueing effect depends on the absolute magnitude of the cue frequency itself, in light of unpublished data from our laboratory, we think it is more likely that the singleton-nonsingleton difference (i.e., between the cue frequency and the 1 Hz nonsingletons) accounted for the increasing cueing effect (our subsequent results show that a low-frequency singleton among high-frequency nonsingletons also leads to maximal attraction of attention). Cass et al. (2011) have reported similar results, demonstrating highly efficient search for flicker frequency differences larger than 5 Hz. However, we also found that the cueing effect increased with cue duration. This is different from abrupt onset cues that already lead to inhibition of return in conditions corresponding to our long-duration cues (see Klein, 2000, for a review). A possible reason is that to discriminate the motion singleton among the nonsingletons, participants had to integrate information across time. Yet, it is also possible that a more general principle explained this duration-dependency, as past research has sometimes found that bottom-up capture in general (e.g., by color singletons) can also benefit from longer singleton durations (Kiss, Grubert, Petersen, & Eimer, 2012). Related to this point, in the present study, by randomizing maximum flicker amplitudes across all stimuli in the cue display, temporal contrast and frequency were unconfounded. Still, however, frequency singletons may have appeared brighter overall than nonsingletons due to increased contrast sensitivity at higher frequencies as defined by the human temporal contrast sensitivity function (tCSF) which peaks around 8 Hz–10 Hz (Bartley, 1939; Hess & Snowden, 1992). Future research should address the question if perceived luminance can also lead to bottom-up capture, with extended distractor durations. Hitherto most studies found no consistent evidence for bottom-up capture by lightness alone (cf. Folk et al., 1992).

## Supplementary Information

ESM 1 (DOCX 118 kb)

## Footnotes

1 Note that in both trial types, invalid and neutral, the target was always preceded by a neutral cue frequency (1 Hz).
