# Supplementary material for: Automatic capture of attention by flicker
Source: Atten Percept Psychophys. 2021 Feb 19;83(4):1407–15. doi: 10.3758/s13414-020-02237-2 (PMC8084820; doi:10.3758/s13414-020-02237-2)

Supplementary Material

***Table S1.*** *Mean RTs (for Correct Trials; ms) in Each Condition of Experiments 1 and 2.*

|  | **Valid Cue** | | | | | | **Invalid Cue** | | | | | | **Neutral Cue** | |
| --- | --- | --- | --- | --- | --- | --- | --- | --- | --- | --- | --- | --- | --- | --- |
| Cue duration (ms) | **180** | | | **380** | | | **180** | | | **380** | | | **180** | **380** |
| Cue Frequency (Hz) | **5** | **10** | **15** | **5** | **10** | **15** | **5** | **10** | **15** | **5** | **10** | **15** | **1** | **1** |
| Experiment 1  singleton search | 619 | 623 | 621 | 627 | 618 | 592 | 647 | 657 | 654 | 642 | 666 | 662 | 645 | 645 |
| Experiment 2  non-singleton search | 705 | 716 | 673 | 705 | 682 | 666 | 716 | 722 | 715 | 715 | 725 | 719 | 713 | 711 |

**Table S2.** *Mean Accuracy (Proportion Correct) in Each Condition of Experiments 1 and 2.*

|  | **Valid Cue** | | | | | | **Invalid Cue** | | | | | | **Neutral Cue** | |
| --- | --- | --- | --- | --- | --- | --- | --- | --- | --- | --- | --- | --- | --- | --- |
| Cue duration (ms) | **180** | | | **380** | | | **180** | | | **380** | | | **180** | **380** |
| Cue Frequency (Hz) | **5** | **10** | **15** | **5** | **10** | **15** | **5** | **10** | **15** | **5** | **10** | **15** | **1** | **1** |
| Experiment 1  singleton search | .97 | .96 | .98 | .97 | .97 | .97 | .96 | .97 | .97 | .96 | .96 | .95 | .97 | .97 |
| Experiment 2  non-singleton search | .96 | .96 | .96 | .97 | .96 | .96 | .96 | .95 | .95 | .97 | .96 | .96 | .96 | .96 |

***Table S3.*** *Mean Cueing Effect (Invalid – Valid RTs) in Each Condition of Experiments 1 and 2.*

|  | **Cueing Effect** | | | | | |
| --- | --- | --- | --- | --- | --- | --- |
| Cue duration (ms) | **180** | | | **380** | | |
| Cue Frequency (Hz) | **5** | **10** | **15** | **5** | **10** | **15** |
| Experiment 1  singleton search | 28.3 | 33.3 | 33.4 | 15.3 | 47.5 | 70.3 |
| Experiment 2  non-singleton search | 11.5 | 6.6 | 41.5 | 9.7 | 43.0 | 53.5 |

***Table S4.*** *Mean Cueing Cost (Invalid – Neutral RTs) and Mean Cueing Benefit (Neutral – Valid RTs) in Each Condition of Experiments 1 and 2.*

|  | **Cueing Cost** | | | | | | **Cueing Benefit** | | | | | |
| --- | --- | --- | --- | --- | --- | --- | --- | --- | --- | --- | --- | --- |
| Cue duration (ms) | **180** | | | **380** | | | **180** | | | **380** | | |
| Cue Frequency (Hz) | **5** | **10** | **15** | **5** | **10** | **15** | **5** | **10** | **15** | **5** | **10** | **15** |
| Experiment 1  singleton search | 2.2 | 11.8 | 9.3 | -2.4 | 20.8 | 17.3 | 26.0 | 21.6 | 24.2 | 17.7 | 26.8 | 53.0 |
| Experiment 2  non-singleton search | 3.4 | 9.1 | 1.6 | 3.4 | 13.2 | 8.0 | 8.2 | -2.5 | 39.9 | 6.33 | 29.8 | 45.5 |

***Figure S1.*** Experiment 1 – singleton search: mean cueing effect, cueing cost and cueing benefit. Error Bars are ±1 *SE.*


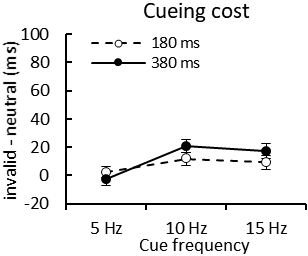

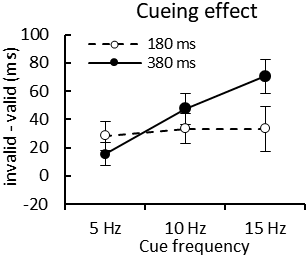

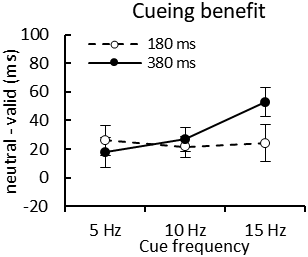


***Figure S2.*** Experiment 2 – non-singleton search: mean cueing effect, cueing cost and cueing benefit. Error Bars are ±1 *SE.*


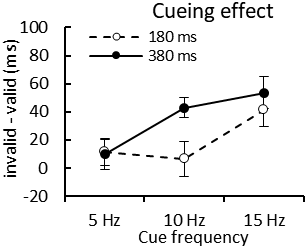

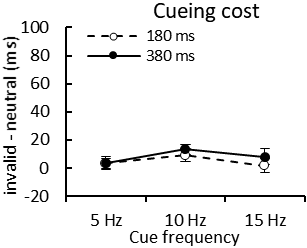

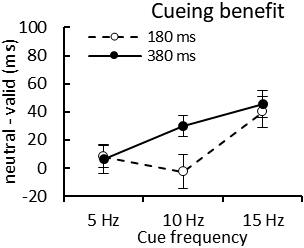

Supplement: Supplementary file 1 — (DOCX 118 kb) [file 13414_2020_2237_MOESM1_ESM.docx]
